# Gross Measurement of Intraarticular Graft Length in All-Inside Anterior Cruciate Ligament Reconstruction

**DOI:** 10.7759/cureus.35964

**Published:** 2023-03-09

**Authors:** Nikolaos E Koukoulias, Angelo V Vasiliadis, Evangelia Germanou, Theodoros M Kannas, Thefilos Dimitriadis

**Affiliations:** 1 Sports Trauma and Orthopaedic Department, St. Luke’s Hospital, Thessaloniki, GRC; 2 Department of Physical Education and Sport Science, Aristotle University of Thessaloniki, Thessaloniki, GRC; 3 Department of Physical Education and Sport Science, Aristotle University of Thessaloniki, Serres, GRC

**Keywords:** knee arthroscopy, graft bottoming out, all- inside technique, intraarticular graft length, anterior cruciate ligament reconstruction

## Abstract

Intra articular length (IAL) of the graft has not been measured yet in anatomic, single bundle, anterior cruciate ligament (ACL) reconstruction. Especially in the all-inside ACL reconstruction technique, the IAL of the graft is of great importance due to the philosophy of the technique and the risk of graft “bottoming out”. We present a simple arthroscopic measurement of the IAL of the ACL graft in anatomic, single bundle ACL reconstruction, that will allow optimal application of the all-inside technique.

## Introduction

The main characteristics of the all-inside anterior cruciate ligament (ACL) reconstruction technique are the use of short grafts and the constructions of sockets, as opposed to full-length tunnels. Historically, it was Morgan et al. [1] that described this technique in 1995. However, the all-inside ACL reconstruction technique was popularized by Lubowitz et al., who introduced the retrograde drilling in 2006 [2] and the outside-in femoral drilling along with the adjustable suspensory cortical fixation in 2011 [3].

The most important risk of this technique is the graft-tunnel mismatch that would lead to inadequate graft tension and as a result to a loose graft. In detail, if the graft length is not less than the sum of femoral socket length plus intra-articular graft distance plus tibia socket length, then “bottoming-out” of the graft will take place and the clinical result of the ACL reconstruction may be compromised [2,3].

As a result, the intra articular length (IAL) of the graft is of great importance for an optimal all-inside ACL reconstruction. Nevertheless, IAL measurement of the graft in anatomic, single bundle ACL reconstruction has not been reported yet. Lubowitz [2] proposed that IAL of the graft may be estimated to range from approximately 29 to 36 mm, depending on the size of the patient and the knee joint. However, anatomy studies have shown that the intact ACL has a mean length of 38 mm (range, 22 to 41 mm) [4].

The purpose of this technical report is to present a simple arthroscopic measurement of the IAL of the ACL graft in anatomic, single bundle ACL reconstruction.

## Technical report

**Figure 1:**
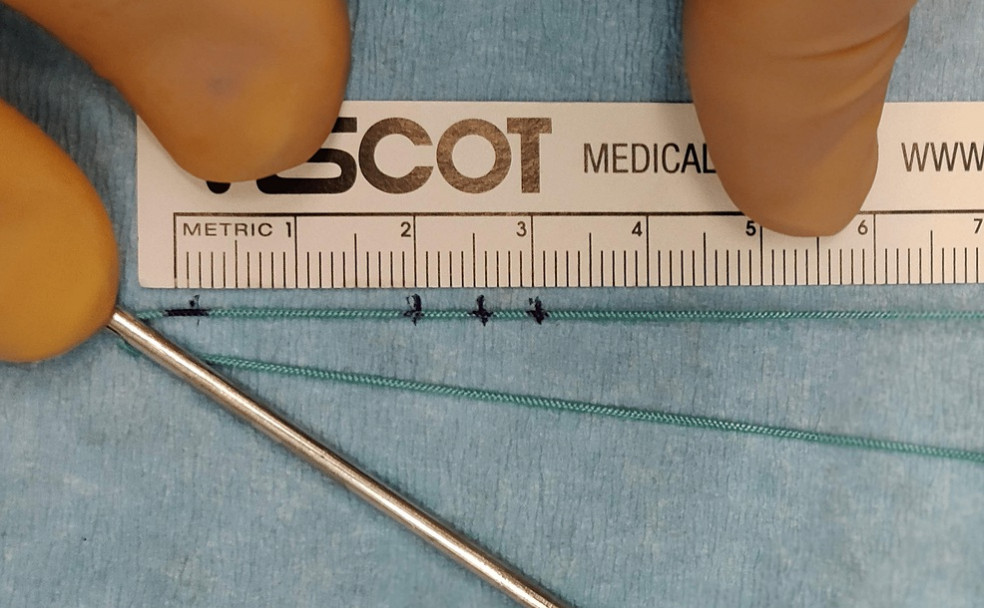
Marking of the shuttle suture.

The femoral socket is drilled with the outside-in technique with the knee flexed at 90°. The anteromedial portal serves as the viewing portal and the outside-in guide is placed in the femoral footprint of the ACL through the anterolateral portal. Once the femoral socket has been constructed with the outside-in technique, a shuttle suture is advanced into the joint with the aid of a pin with an eyelet, that will later help in graft passage. The shuttle suture has been previously marked to serve as a ruler. In detail, the center of the length of the suture is marked with a marking pen. This mark will be the zero-point. Additional marks are then made at a distance of 20, 25 and 30 mm (Figure 1).

**Figure 2:**
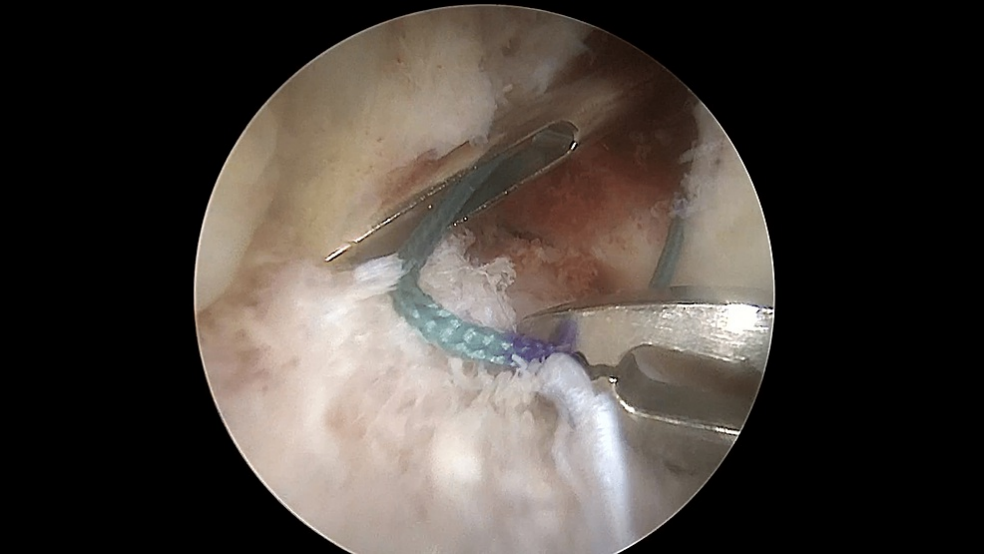
Left knee. The antero-medial portal is the viewing portal. The shuttle suture is introduced into the joint and grasped with a grasper (inserted through the antero-lateral portal) from the zero-point mark. Arthroscopic view.

The marked shuttle suture is grasped with a KingFisher grasper (Arthrex, Naples, FL, USA) from the zero-point mark (Figure 2).

**Figure 3:**
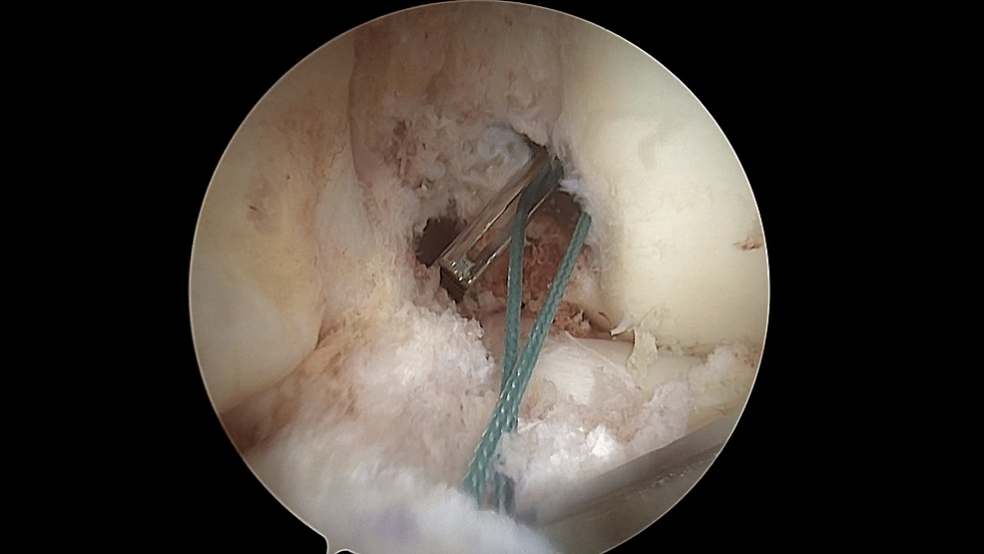
Left knee. The antero-medial portal is the viewing portal. Measurement of the intra articular length of the graft. Arthroscopic view.

The shuttle suture is then brought to the center of the ACL tibia footprint, put under slight tension, in order not to be loose, and the guide pin is moved backwards so that the base of the eyelet is flush with the level of the femoral socket entrance (Figure 3).

Since the guide pin is in the center of the femoral socket, the shuttle suture that rests at the base of the guide pin eyelet is positioned in the center of the femoral ACL footprint.

The length of the shuttle suture at this point represents the intra-articular distance of the ACL graft. The surgeon reads the 20, 25 and 30 mm marks on the shuttle suture and has a gross IAL graft measurement at 90° of knee flexion.

## Discussion

**Figure 4:**
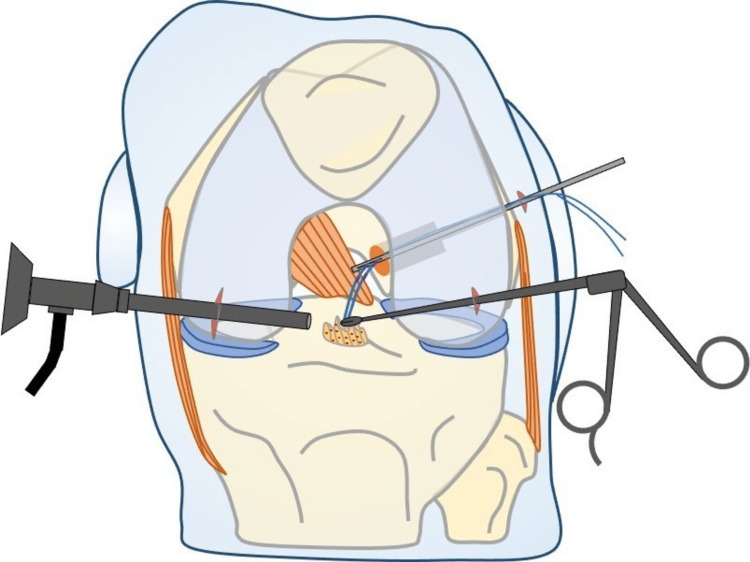
Drawing of the technique.

Our technique (Figure 4) is the first that reliably evaluates the real IAL of the ACL graft intraoperatively, because the measurement is based on the centers of the femoral and tibial footprints. So far, the IAL of the ACL graft has been measured in the transtibial technique with a ruler that was introduced through the full-length tibial tunnel [5]. This technique is not applicable in the all-inside ACL reconstruction technique, because sockets instead of full-lengths are used. Moreover, in the transtibial measurement technique, the ruler crosses neither the centers of the ACL femoral and tibial footprints nor the centers of tibia and femoral tunnels and as a result, the measurement is neither anatomic nor accurate.

**Figure 5:**
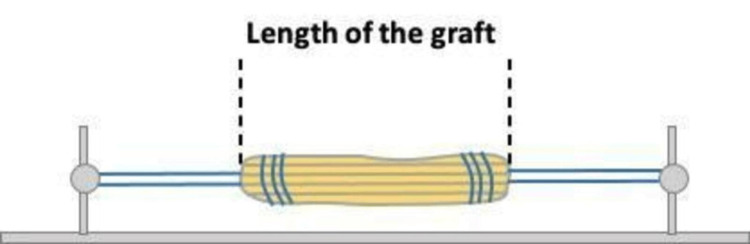
Drawing of the length of the graft after tensioning.

IAL graft measurement is very important in the all-inside ACL reconstruction technique. The length of the graft (GL) after tensioning (Figure 5), has to be less than the sum of the femoral socket (FS) and tibial socket (TS) plus the IAL of the graft. In this equation (GL<FS+TS+IAL), the only unknown factor is the IAL of the graft. According to Lubowitz [2] who described the technique, the IAL of the graft should be estimated to be 29 to 36 mm. Based on that assumption and knowing the length of the graft, the surgeon decides the length of the femoral and tibial socket drilling.

**Figure 6:**
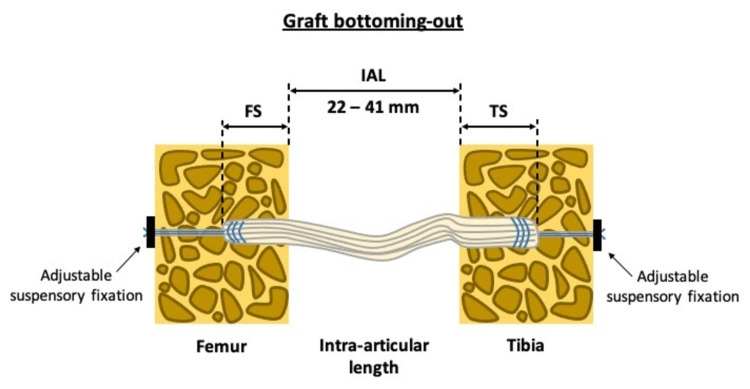
Drawing of the factors affecting the graft “bottoming-out”. FS: femoral socket, TS: tibial socket, IAL: intra articular length of the graft

**Figure 7:**
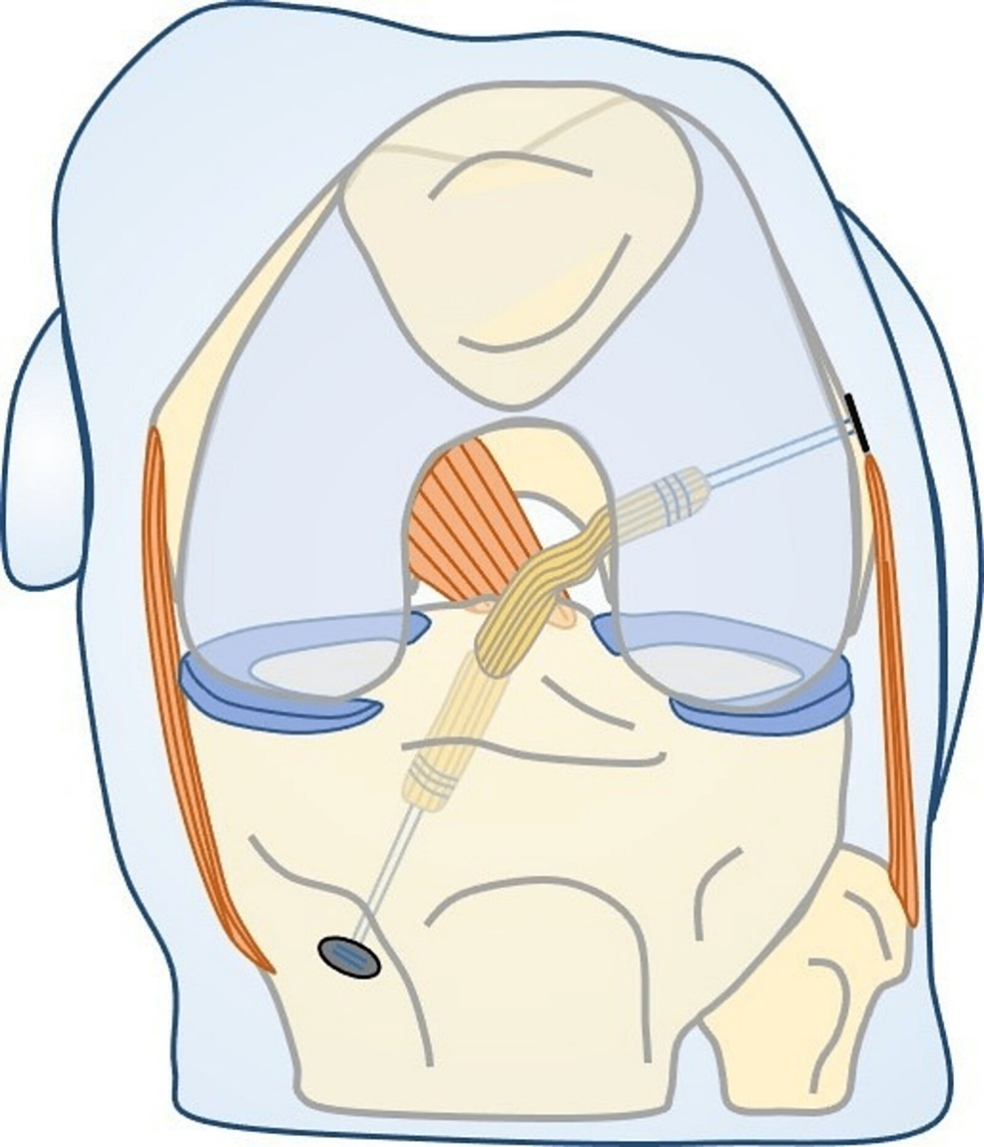
Drawing of clinical appearance of ACL graft “bottoming-out” (loose graft). ACL: anterior cruciate ligament

In case of underestimation (IAL of the graft more than estimated), the graft cannot be tensioned because of “bottoming-out” and the graft remain loose (Figures 6, 7).

In this scenario, the surgeon should remove the graft and recreate the sockets, increasing their length. This is a difficult task to accomplish, and it may necessitate removal of the hardware used for graft fixation (buttons) and utilization of new hardware. As a result, graft bottoming out needs additional surgical time and risk and may necessitate increased surgical exposure and cost.

**Figure 8:**
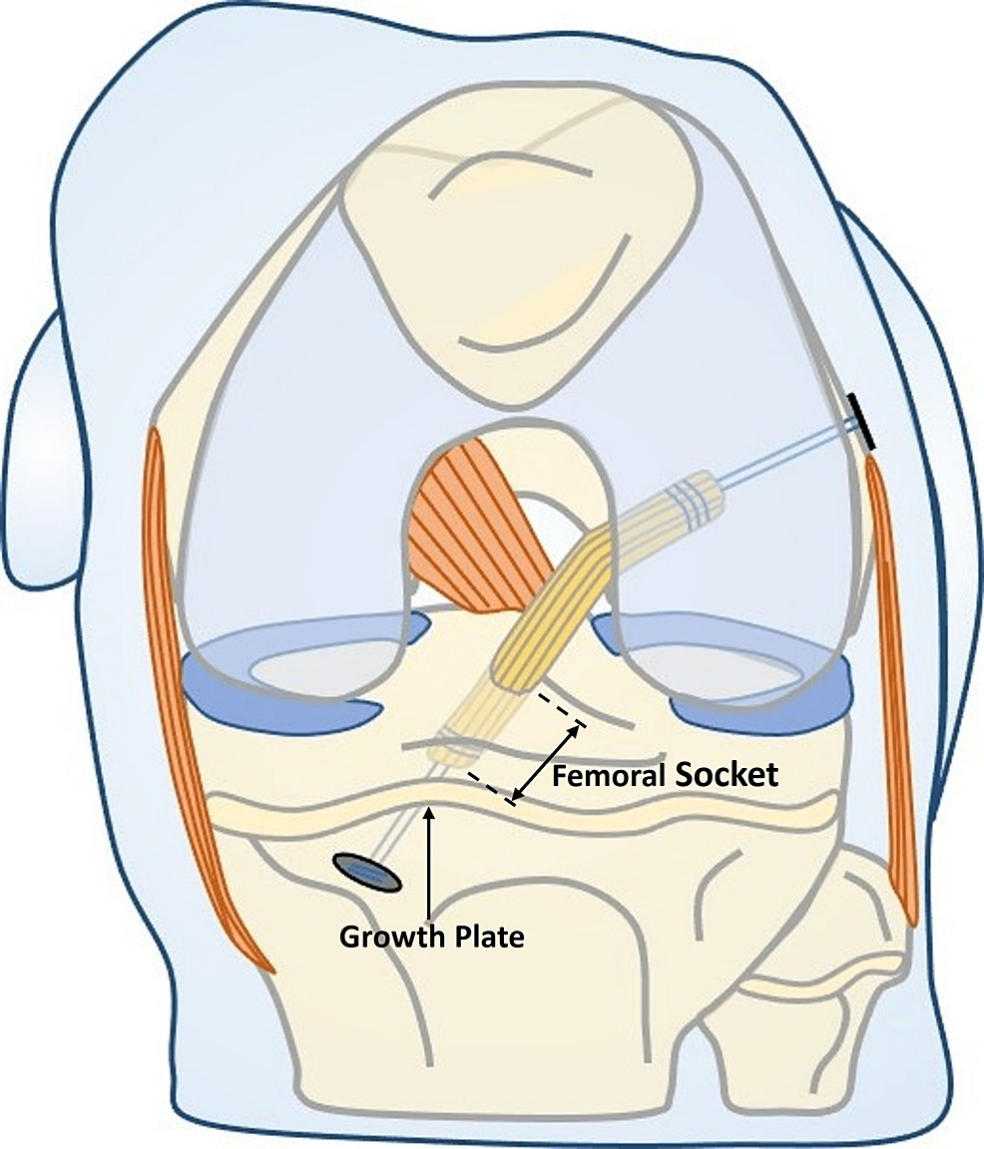
In paediatric ACL reconstruction, the femoral socket length has an upper limit, determined by the growth plate. ACL: anterior cruciate ligament

In case of overestimation (IAL of the graft less than estimated), the surgeon will drill longer than needed sockets. Such a scenario could make non-applicable the use of the all-inside ACL reconstruction technique in small knees, where small in-length sockets are needed to accomplish the technique. Especially in paediatric ACL reconstruction, where full epiphyseal sockets are mandatory in order to avoid growth disturbance, the length of the sockets has an upper limit, determined by the growth plate (Figure 8).

Moreover, the utilization of sockets offers the advantage of tunnel enlargement prevention and graft maturation acceleration, due to the dead space eradication [6]. As a result, the unneeded lengthening of the sockets deprives one of the advantages of the all-inside ACL reconstruction technique.

The length of the ACL has been found to be influenced by patient’s sex and height and has a wide range of 20 mm (22 to 41 mm) [5,7]. This broad range of IAL graft length does not permit individualized ACL surgery with assumptions. Moreover, the concept of all-inside ACL reconstruction requires as precise as possible IAL graft measurement.

Our technique measures the IAL of the graft at 90° of knee flexion. We have, though, to underline that anatomic, single bundle ACL reconstruction results in different IAL of the graft at 90° and full knee extension. Yoo et al. [8] used a three-dimensional computer tomography system to assess the length changes of the virtual anterior cruciate ligament and showed that the length of the ACL in flexion is 2.82 ± 0.14 cm, while the length of the ACL in full extension is 3.49 ± 0.22. Since our technique cannot be used with the knee in full extension due to lack of visibility, this difference should be taken into account by the surgeon when planning the socket construction.

Another limitation of our technique is the fact that it gives only a gross measurement of the IAL of the graft. The marking of the shuttle suture cannot indicate values of one mm accuracy since the ink is absorbed by the suture. Nevertheless, the surgeon can easily recognize whether the IAL graft is 20 mm, or near 20 etc. In our opinion the construction of a calibrated shuttle suture would overcome this limitation and our technique would give an accurate result.

## Conclusions

In conclusion, intra articular graft length measurement is of great importance in all-inside ACL reconstruction, since it determines the length of the sockets. Short sockets may lead to graft bottoming out while long sockets eliminate the advantages of the all-inside technique or make it non-applicable. Our technique of intra articular graft length measurement is currently the only accurate and anatomic available technique.
